# Plant Kin Recognition Enhances Abundance of Symbiotic Microbial Partner

**DOI:** 10.1371/journal.pone.0045648

**Published:** 2012-09-28

**Authors:** Amanda L. File, John Klironomos, Hafiz Maherali, Susan A. Dudley

**Affiliations:** 1 Department of Biology, McMaster University, Hamilton, Ontario, Canada; 2 Department of Biology, University of British Columbia, Kelowna, British Columbia, Canada; 3 Department of Integrative Biology, University of Guelph, Guelph, Ontario, Canada; Centro de Investigación y de Estudios Avanzados, Mexico

## Abstract

**Background:**

The stability of cooperative interactions among different species can be compromised by cheating. In the plant-mycorrhizal fungi symbiosis, a single mycorrhizal network may interact with many plants, providing the opportunity for individual plants to cheat by obtaining nutrients from the fungi without donating carbon. Here we determine whether kin selection may favour plant investment in the mycorrhizal network, reducing the incentive to cheat when relatives interact with a single network.

**Methodology/Principal Findings:**

We show that mycorrhizal network size and root colonization were greater when *Ambrosia artemisiifolia L*. was grown with siblings compared to strangers. Soil fungal abundance was positively correlated with group leaf nitrogen, and increased root colonization was associated with a reduced number of pathogen-induced root lesions, indicating greater benefit to plants grown with siblings.

**Conclusions/Significance:**

Plants can benefit their relatives through investment in mycorrhizal fungi, and kin selection in plants could promote the persistence of the mycorrhizal symbiosis.

## Introduction

Many organisms cooperate even though they have the opportunity to cheat. The interaction between plants and mycorrhizal fungi is considered a mutualism because the fungus provides water, nutrients and pathogen defense to the plant in return for carbohydrates. Though most mycorrhizal fungi are obligate symbionts, dependent on plant carbon for growth [1], plants may be obligate or facultative in their association with mycorrhizal fungi [2]. Moreover, mycorrhizal fungi may span the gradient from mutualism to parasitism. Cooperation, conflict, and cheating have all been observed to occur between fungi and plants [3], [4].

The symbiosis is considered by economic models to be a biological market where there is a trade relationship between plant and fungi, each of which specializes on acquiring certain resources [5]–[7]. Models show that a mutualism can be stable through a trade relationship [5], [6]. Plants tend to associate more with mycorrhizas when soil nutrients (*e.g.* [8], [9]) or plant tissue phosphorus (P) concentration [10] are low, which supports a simple prediction from the biological market models. Recent experimental evidence indicates that, given a choice, plant and fungal partners can also choose to trade with more cooperative partners, thus promoting a stable mutualism where neither partner is in control of the other [11].

When many plants are connected to a common mycorrhizal network (CMN), tragedy of the commons theory models the mycorrhizal symbiosis as a social good, *i.e.*, a common good that is a shared resource created and/or maintained by the group [12]. For mycorrhizas, the CMN may be maintained by a group of plants and provides a common resource for that group. The size of the fungal network depends on plant carbohydrate contributions and thus, more soil colonization by fungal hyphae implies more investment by the plant partner [13]. Therefore, the value of the mycorrhizal network as a social good should depend on the summed carbon donations from host plants. Because attached plants will acquire more nutrients from larger networks with greater surface area and increased soil exploration, plants benefit each other by investing in the same fungal partner.

However, as individuals pay a cost to participate in the symbiosis, this creates a conflict. If individuals can escape paying the cost while still reaping the benefits from their partner, there is strong incentive to cheat [14]. In the mycorrhizal symbiosis, several plants may be attached to a CMN and many fungal genets or species can simultaneously colonize a single plant. If either the fungus or plant do not identify cheaters and invoke sanctions, the symbiosis is open to non-cooperators since individuals may attach themselves to the mutualism without donating their fair share, ultimately leading to a tragedy of the commons [12], [15]–[17]. A majority of research has concentrated on the potential role of sanctions against cheaters [18]–[20]. However, kin selection among plants offers an alternate incentive for cooperation between mutualists [21], [22] because for a plant, investing carbon in the mycorrhizal network linked to close relatives could increase one's indirect fitness and may remove cheaters from the population [23] preventing a tragedy of the commons [12].

Plants frequently live in dense communities where relatedness may be high, providing the opportunity for kin selection [24], [25]. Kin selection acts more strongly if individuals only demonstrate altruism toward relatives [26], which then favours the evolution of kin recognition. Kin recognition has been demonstrated in several species of plants [27]–[30]. Though the mechanism is as yet unknown, root exudates have been demonstrated to convey a signal [27]. Kin recognition is also manifested as phenotypic plasticity in resource-gathering structures in response to relatedness of the plant group. In *Cakile edentula*, for example, allocation to fine roots was lower among individuals in sibling groups [28] relative to groups of non-related individuals. Because fine roots are the sites of nutrient and water absorption, this response suggests that competition for these resources was reduced among siblings (*i.e.,* kin). However, these studies demonstrating kin recognition have been done using non-mycorrhizal plants, and it is possible that the presence of a symbiont could influence interactions among kin.

Although researchers have considered the importance of plant neighbourhood on mycorrhizas, these studies have focused on the benefits of fungal [31] and plant diversity [32]–[36]. In the only study that has tested whether the genetic relatedness of neighbours influenced plant interactions with mycorrhizas, Ronsheim & Anderson (2001) found that in the presence of soil fungi, biomass of individuals grown with clones or plants from the same population was greater than individuals grown with plants from a different population [37]. Their study addressed the question of local adaptation to soil fungal communities and they demonstrated benefits of growing with plants from the same population. However, no study has yet measured kin recognition in mycorrhizal plants or tested whether relatedness of a plant population affects mycorrhizal fungal growth. When mycorrhizas are present, greater cooperation among groups of siblings could be manifested through an increase in the CMN. Such an increase could result in greater total nutrient acquisition for the group [38] or reduce the likelihood of pathogen attack [39], which should enhance the fitness of groups of siblings relative to groups of strangers.

We examined whether the association between *Glomus intraradices* and pairs of *Ambrosia artemisiifolia L.* (common ragweed) seedlings depended on the relatedness of the two plants. *G. intraradices* colonizes plant roots aggressively [40], suggesting that young plants may experience kin selection through mycorrhizas. Because arbuscules are the sites of nutrient exchange and an increase in root colonization by arbuscules indicates a well-established mutualism [41], we predicted that plant kin selection would favour the colonization of arbuscules in sibling pairs. To determine whether related seedlings benefited from a potentially enhanced mycorrhizal association, we measured plant growth as well as susceptibility to pathogen attack by measuring the frequency of lesions on roots.

Since an increase in mycorrhizal association in young seedlings may promote a well-developed CMN later in life, we carried out a second experiment to investigate whether plant relatedness and P level affected the symbiosis at the juvenile stage, when the CMN has had time to develop. Hyphae from spores of the same isolate of *G. intraradices* readily fuse together [42], increasing the likelihood of a CMN forming. We predicted that kin selection would favour siblings to donate more carbon to the fungal partner, resulting in greater mycorrhizal association in groups of siblings than in groups of strangers. We also predicted that plants would promote mycorrhizal colonization in lower P environments, where the symbiosis could facilitate plant nutrient acquisition, regardless of the relatedness of the group. We examined whether an enhanced CMN, quantified as the length of the extraradical mycorrhizal hyphae, benefitted plants by measuring the relationship between CMN size and plant growth, as well as between CMN size and leaf nitrogen (N).

We present results that show the mycorrhizal association meets two predictions supported by kin selection theory: plants grown in siblings groups had more mycorrhizal colonization and growth than when they are grown in stranger groups, and the increased mycorrhizal association benefits the plants. Seedlings grown with siblings had more arbuscules and root hyphae and a reduced proportion of lesions on the roots. Juveniles had longer soil hyphae when grown with siblings, suggesting a more developed CMN, and this was correlated with increased leaf N. We also found that stranger groups had longer soil hyphae in low P, but soil hyphal length and growth was promoted in sibling groups regardless of P level. Alternative hypotheses for these results were explored but these hypotheses were not supported.

## Materials and Methods

*A. artemisiifolia L.* is a fast growing, wind-pollinated annual plant that readily associates with mycorrhizal fungi, and *G. intraradices* is a widely-distributed arbuscular mycorrhizal fungus (AMF) that has positive effects on ragweed performance [43]. Two greenhouse experiments were conducted at separate times. For both experiments, field pollinated seeds from maternal sibships (families) were stratified on moist sand at 4°C for three weeks. We transplanted to pots containing a soil-free mixture of 3∶1 sand and Turface (Profile Products LC, Buffalo Grove, IL, USA) 4 days after germination for experiment 1 and 8 days after germination for experiment 2. Turface is a calcined clay product. A mix of turface and sand provides a substrate that drains well, releases water slowly, and readily separates from roots. Though we did not sterilize the growth medium, it was mixed from un-opened bags and did not include any type of field soil. Moreover, levels of soil fungal hyphae were marked lower in control compared to inoculated treatments. Plants in experiment 1 were measured at the seedling stage and plants in experiment 2 were measured at the juvenile (pre-reproductive) stage.

### Experiment 1 (seedlings, pairs)

To test the prediction that social environment affects the mycorrhizal association, we conducted a fully factorial experiment with the following treatments: social environment (siblings vs. strangers) and mycorrhizas (inoculated vs. un-inoculated). At this early life-stage, the mycorrhizal hyphal network is not yet established in the soil but plants are colonized by various fungal structures including arbuscules, the sites of nutrient exchange. Each pair of plants was grown in an 8.9 cm diameter, plastic pot. Six families were used to manipulate the social environment with either two siblings (same family) or two strangers (different families) per pot. Fifteen possible stranger combinations were replicated four times, and the six sibling pairs were replicated ten times across the experiment. Half the pots were inoculated with a commercially available product containing spores of *G. intraradices* mixed with a sterile media (30 mL/pot, Myke Annual and Perennial, Premier Tech Biotechnologies, Riviere-du-Loup, QC), spread onto the sand/turface, approximately 2.5 cm below the soil surface, prior to transplanting. Half the pots remained un-inoculated. Because we did not add un-inoculated media to non-mycorrhizal pots to control for the effect it might have on soil structure and therefore root growth, we were only able to compare belowground plant traits within mycorrhizal treatments.

The experiment was arranged into six blocks, each of which contained 20 randomly arranged pots from all possible treatment combinations. In total, 240 plants were grown in the greenhouse for 4 weeks under natural and supplementary light. Blocks were randomly rearranged on the bench every week. All plants were given a weekly dose of low P fertilizer (831 ppm, 21-5-20 NPK, Peter's Excel, Scott's Company, Marysville, OH, USA) in solution until the soil was saturated.

Four weeks after transplantation, plants were harvested above- and belowground. Leaves and stems for each plant were dried to constant mass at 37.8°C and weighed. A sample of roots and soil was taken from the bottom 2 cm of the pot. Half of this sample was used for fungal quantification and measurement of root lesions and the other half was washed for root biomass estimation. The rest of the roots in the pot were washed clean of substrate, dried and separated into fine roots (<1 mm) and coarse roots (>1 mm). Root biomass was quantified as the total from both plants in each pot since it was not possible to identify roots from either plant. Due to the destructive nature of washing roots, root morphological traits were not measured. Mycorrhizal fungi were quantified as percent of the root colonized by arbuscules, vesicles and hyphae. Soil hyphal length was not measured for this experiment because there was not enough time for sufficient soil hyphal colonization. Fungal colonization data used for analysis was the average of two samples taken from each pot. No AMF were found in the un-inoculated pots, confirming that our soil did not contain mycorrhizal fungi and there was no cross-contamination across treatments.

Mycorrhizal fungi are known to protect roots from pathogens and other enemies. We assessed the benefit of mycorrhizal colonization for seedlings as the percent of the root affected by lesions. There was no intentional addition of pathogens to the soil for our investigation of the protective effect of the mycorrhizas. Thus, any lesions found on the roots were the result of airborne pathogens commonly found in a greenhouse setting. An observer who was double blind to treatments quantified lesions. The observer counted any damage sites on the plant roots as a lesion regardless of source because we were interested in the general protective effect mycorrhizas have against lesions, not specific pathogens.

### Experiment 2 (juvenile, groups of four)

To test the prediction that older sibling plants grown with mycorrhizas would also increase their association with the fungal partner compared to strangers and to test for mycorrhizal and plant responses to P level, we conducted a second fully factorial experiment that included the following treatments: social environment, mycorrhizas, and P level.

For the social environment treatment, four maternal sibships (families) were used to manipulate the relatedness of each group; either four siblings (same family) in a large (7.3 cm×7.6 cm×35.6 cm) pot, four strangers (four different families) in a large pot, or four solitary plants, one from each family, in their own smaller pots (3.8 cm×3.8 cm×35.6 cm). Pots were open-ended cellulose bands (Zipset plant bands, Monarch Manufacturing, Colorado), which have a longer rooting depth than the plastic pots used in experiment 1, making them ideal for a longer-term study. To prevent growth of saprobes, a common problem when using these pots, they were soaked in fungicide and dried prior to experimental set up.

To manipulate P level, half the juvenile plants were given high P (3 g/plant, 14-14-14 NPK, Smartcote, Spectrum Brands IP, Brantford, Ont.) and half with relatively low P fertilizer 3 g/plant, 15-10-15 NPK, Haifa Multicote, Haifa Chemicals Ltd, FL, USA). Control release fertilizer (CRF) was applied on the substrate surface and gradually dissolved with each watering. This method was used because of the difficulty of applying nutrients in solution consistently after the canopy closes in high-density stands. Our manipulation of P level was not extreme since we designed the study to investigate plant-plant interactions and plant-fungal interactions rather than response to nutrient stress.

For the mycorrhizal treatment, half the groups were inoculated with spores of a single isolate of *G. intraradices* in solution (50 spores/mL, 10 mL/plant) and the other half were not. Spores were spread onto a layer of compost soil, 5 cm from the top of the substrate surface. This layer of compost was covered with sand/turface to fill the pot. The un-inoculated groups also had the layer of compost but no spores were applied. This allowed us to control for the effect the compost may have on substrate structure, which could affect root growth. Inoculated and un-inoculated pots were randomly arranged within blocks, touching each other. Although AMF colonized roots of inoculated plants (Fig S1), no AMF were found colonizing the roots of un-inoculated plants, indicating no cross contamination of fungal spores from inoculated pots. The inoculation protocol in the juvenile experiment differed from that in the seedling experiment because we were able to acquire cultured spores of *G. intraradices*, which allowed more precise control of the number of spores applied to each plant.

The entire experiment consisted of six blocks with at least 30 replicates of each possible treatment combination. Each tray contained 16 pots and two trays were combined to create a block containing 32 randomly arranged groups of four from all possible treatment combinations. Plants were watered every second day until pots were saturated. In total, 768 plants were grown in the greenhouse under natural and supplementary light. Any seedlings that died within the first three days of transplanting were replaced.

Plants were harvested after 15 weeks of growth, at the juvenile stage. At this point, the soil hyphal network had time to develop and was measured in meters of hyphae per gram of soil. The soil hypha is the fungal structure used to forage for nutrients and consequently, the size of the hyphal network is a strong predictor of nutrient uptake in mycorrhizal plants [44]. Because mycorrhizal fungi are obligate biotrophs, carbon from the plant partner is required for soil hyphal growth and hyphal length is therefore a metric of plant investment. *G. intraradices* has been shown to produce relatively high numbers of vesicles and intra-radical hyphae and low levels of soil hyphae compared to other mycorrhizal fungi [39]. However, a previous study suggests there is no trade-off between fungal structures [44], which may otherwise confound an effect of plant investment on hyphal length.

After harvest, leaves and stems were dried to constant mass at 37.8°C and weighed for each plant. Before roots were cleaned of substrate, a sample of roots and soil was taken from the bottom 2.5 cm of the pot. Half of this sample was used for fungal quantification and the other half was washed for root biomass estimation. Once cleaned of substrate, roots were dried to constant mass at 37.8°C and separated into fine roots (<1 mm) and coarse roots (>1 mm). They were quantified as the total from a large pot or the sum of four solitary pots. Root morphological traits were not measured. Mycorrhizal fungi were quantified as percent of the root colonized by arbuscules, vesicles and hyphae, and soil hyphal length (m/g soil). An observer who was double blind to treatments carried out fungal quantification.

Leaf N concentration was analyzed for a subset of pots (*n* = 40) given low P, on a 500 – 700 mg sub-sample through dry combustion (900°C) using the Variomax CN Elemental Analyzer (Elementar Americas, Inc., Mt. Laurel, NJ). We analyzed leaf N rather than P because of the cost associated with analyzing an appropriate number of samples for statistical analysis and the availability of equipment. Only pots containing three or four plants were included in the analysis. Ten groups from each combination of kin or stranger, inoculated or un-inoculated treatments were sampled, for a total of 40 groups. Leaves from each plant in the pot were mixed together providing a pooled estimate of leaf nitrogen for each pot.

### Statistical analysis

SAS (version 9.2; SAD, Cary, NC, USA) was used for statistical analysis. PROC GLM was used to conduct analysis of variance (ANOVA) and covariance (ANCOVA). Biomass variables were log transformed to satisfy the assumptions of GLM. Data presented are the back-transformed least squares means (lsmeans). For aboveground traits of both seedlings and juveniles, the individual was the observation. Because the roots could not be identified to an individual, the pair of two plants (seedling experiment) and the group of four plants (juvenile) was the observation for root, fungal, and allocation traits. In the seedling experiment, all pots were shared. In the juvenile experiment, we summed the root masses of the group of four solitary plants in order to achieve similar statistical distributions for solitary and shared groups. For biomass allocation, we summed the aboveground masses of the group of plants. For analysis of fungal traits, our null hypothesis for the juvenile experiment was that the measures of fungal colonization for the mix of roots in a large pot would be equivalent to the average of four plants of the same genotypes in solitary pots.

#### Seedling experiment

To test for effects of treatments on arbuscule, vesicle and hyphal root colonization, ANOVA was done for pairs of inoculated plants only because no fungal structures were found on un-inoculated plants. Here, block, social environment, social environment × block, and family were the independent variables (Table S1). To test for treatment effects on individual aboveground plant biomass, ANOVA was done with log aboveground biomass as the dependent variable and mycorrhizal inoculation, social environment, family and their interactions and block as independent variables (Table 1). To test for an effect of treatments on lesions, ANOVA was conducted; mycorrhizal inoculation, social environment, family and their interactions and block were independent variables, and lesions measured as a percent of root length was the dependent variable. To test the hypothesis that root colonization differed among maternal families, ANOVAs were conducted on the subset of sibling pairs inoculated with mycorrhizal fungi, with plant maternal family as the independent variable, and arbuscule, vesicle and hyphal root colonization as the dependent variables. Effects of treatments on root allocation were measured with ANCOVA with log combined root mass as the dependent variable and log combined aboveground mass as the covariate (PROC GLM).

**Table 1:** Analysis of variance on aboveground biomass for ragweed seedling pairs.

	Log aboveground biomass (g)		
Source	DF	F	*P*
Social environment	1	0.01	0.9138
Mycorrhizas	1	0.98	0.3244
Family	5	15.67	**<0.0001**
SocialEnv × Myc	1	1.21	0.2683
Myc × Fam	5	0.93	0.4606
SocialEnv × Fam	5	1.20	0.3077
SocialEnv × Myc × Fam	5	1.78	0.1184
Block	1	0.29	0.5911

Log of aboveground biomass is log (abovemass +1). Social environment and mycorrhizas refer to treatments. Block refers to the experimental unit. Family refers to the specific pairing of maternal sibships within each pot. Significant values are in bold.

#### Juvenile experiment

In this experiment the social environment treatment included a relatedness component (kin vs. strangers) and a root-neighbour component (presence or absence of root neighbours). We therefore carried out ANOVA and ANCOVA in PROC GLM and used pre-planned *a priori* contrast statements (kin vs. stranger and solitary vs. shared) when social environment had a significant main effect or interaction effect. This allowed us to distinguish whether the social environment effects were due to relatedness and/or the presence of root neighbours.

To test for the effect of treatments on soil hyphal length, ANOVA was conducted. Soil hyphal length was the dependent variable; block, mycorrhizas, social environment and nutrient treatments were the independent variables (Table 2). We used contrast statements to distinguish between effects of social environment × mycorrhizas and social environment × mycorrhizas × P level interactions (Table 2). Correlation analysis (PROC CORR) was used to examine the relationship between estimated total leaf nitrogen and soil hyphal length for plants in large pots. PROC REG was used to test for a relationship between estimated total nitrogen and soil hyphae in plants in shared pots, inoculated with mycorrhizas. To test whether family had an effect on soil hyphal colonization, ANOVA was done on groups of siblings with soil hyphal length as the dependent variable and maternal family as the independent variable. To test whether root sample size influenced hyphal length, regression analysis was conducted using soil hyphal length as the dependent variable and root sample mass as the independent variable.

**Table 2:** Analysis of variance for groups of four juvenile ragweed plants.

	Soil hyphae		
Source	DF	F	*P*
Social environment	2	52.7	**<0.0001**
Kin vs. stranger	1	95.65	**<0.0001**
Solitary vs. shared	1	12.58	**0.0005**
Mycorrhizas	1	1173.47	**<0.0001**
P level	1	6.10	**0.0146**
SocialEnv × Myc	2	33.79	**<0.0001**
Kin vs. stranger × Myc	1	61.00	**<0.0001**
Solitary vs. shared × Myc	1	8.33	**0.0044**
SocialEnv × P level	2	1.49	0.2282
Myc × P level	1	3.55	0.0612
SocialEnv × M × P	2	5.22	**0.0064**
Kin vs. stranger × M × P	1	9.66	**0.0022**
Solitary vs. shared × M × P	1	0.92	0.3394
Block	5	0.94	0.4568

Social environment, mycorrhizas and P level refer to treatment effects. Where there is a significant effect of social environment in an interaction, PROC GLM with pre-planned contrast statements were used to distinguish between effects of kin vs. stranger and solitary vs. shared. Significant values are in bold.

Plants in un-inoculated pots served as a control and fungal quantification verified that mycorrhizal fungi were absent from these pots. Thus, when analyzing strictly mycorrhizal structures, arbuscules, vesicles and root hyphae, only plants in mycorrhizal pots were included in the analysis. To test for the effect of treatments on arbuscule and vesicle colonization, ANCOVA was conducted using log fine root as the covariate. Fine root mass was chosen as a covariate to control for plants that had more roots possibly having increased root colonization. Block, social environment and P level were the independent variables. To test for the effect of treatments on log aboveground biomass, ANOVA was conducted using contrast statements to analyze social environment × family, social environment × P level and social environment × mycorrhizas × P level interactions (Table 3). PROC CORR was used to examine the relationships among fungal colonization, belowground biomass and aboveground biomass. Effects of treatments on root allocation were measured in ANCOVA with log combined root mass as the dependent variable and log combined aboveground mass as the covariate (PROC GLM).

**Table 3:** Analysis of variance (ANOVA) for individual juvenile ragweed plants.

	Log above mass (g)		
Source	DF	F	*P*
Family	3	7.73	**<0.0001**
Social environment	2	0.03	0.9725
Mycorrhizas	1	0.74	0.3911
P level	1	3.75	0.0532
SocialEnv × Family	6	3.52	**0.0020**
Kin vs. stranger × Fam	3	3.84	**0.0097**
Solitary vs. shared × Fam	3	3.36	**0.0187**
Myc × Family	3	1.95	0.1207
P level × Family	3	3.37	**0.0182**
SocialEnv × Myc	2	0.18	0.8352
SocialEnv × P level	2	4.34	**0.0135**
Kin vs. stranger × P	1	0.18	0.6744
Solitary vs. shared × P	1	8.57	**0.0036**
Myc × P level	1	2.32	0.1279
SocialEnv × M × F	6	0.85	0.5310
SocialEnv × P × F	6	0.62	0.7117
SocialEnv × M × P	2	4.02	**0.0184**
Kin vs. stranger × M × P	1	1.98	0.1600
Solitary vs. shared × M × P	1	5.84	**0.0160**
SocialEnv × M × P × F	6	1.91	0.0766
Block	5	3.23	**0.0070**

Log of aboveground biomass is log (aboveground biomass+0.5). Family refers to maternal sibship. Social environment, mycorrhizas and P level refer to treatment effects. Where there is a significant effect of social environment in an interaction, PROC GLM with pre-planned contrast statements were used to distinguish between effects of kin vs. stranger and solitary vs. shared. Significant values are in bold.

## Results

### Responses in Seedling Pairs

**Figure 1:**
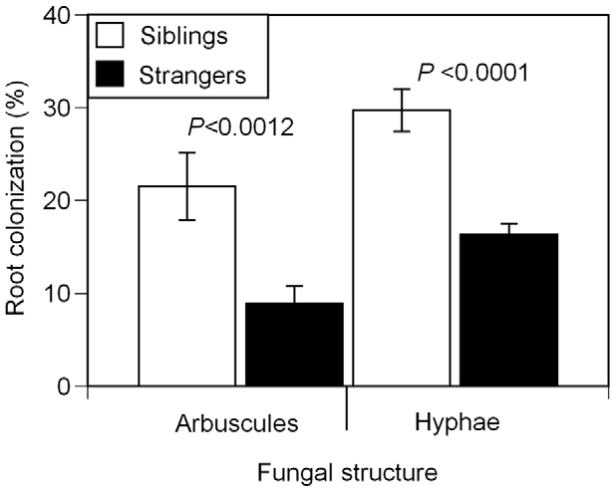
Root colonization by arbuscules and hyphae in *A. artemisiifolia L* seedlings. Ragweed seedlings inoculated with *G. intraradices* were grown in pairs of either siblings (white bars) or strangers (black bars) (*n* = 119). Sibling roots had significantly more arbuscular colonization (*P<*0.0012) and hyphal colonization (*P*<0.0001) compared to stranger roots. Error bars represent ±1 s.e.m.

We found evidence that social environment affects mycorrhizal colonization in seedling pairs, as kin selection would predict. Whether or not seedlings were inoculated, there was no evidence of plants responding to the relatedness of their neighbours in log aboveground biomass (Table 1), stem elongation (Table S2), leaf:stem allocation (Table S3), and root:shoot allocation (Table S4). However, there was an effect of social environment on mycorrhizal root colonization in resource exchange traits; siblings in inoculated pots had 82% more arbuscules and 142% more hyphal colonization compared to strangers (Fig 1, Table S1). There was a significant effect of family on vesicle colonization (Table S1), such that some family combinations had significantly more vesicles than others.

### Responses in Juvenile groups

**Figure 2:**
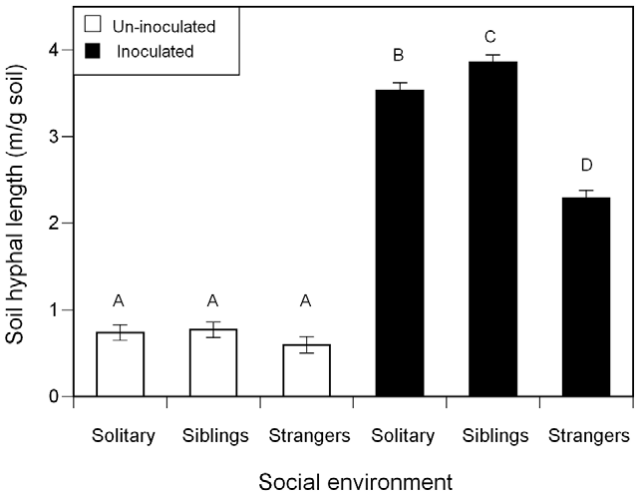
Effect of mycorrhizal inoculation and social environment on soil hyphal length for juvenile *A. artemisiifolia L* plants. Groups of four plants were either un-inoculated (white bars) or inoculated with *G. intraradices* (black bars). Plants were grown solitary, with siblings or with strangers. Soil hyphal length was lower in un-inoculated plants and did not differ among social environments; however, soil hyphal length differed markedly among social environments in inoculated groups (social environment × mycorrhizas interaction *P*<0.0001). Log fine root mass was included as the covariate but had no significant effect. Means that did not differ significantly at P<0.05 are represented by the same letter. Error bars represent ±1 s.e.m.

Though there was no evidence for juvenile plants responding to the relatedness of their neighbours in biomass (Table 3) and morphology, we did find neighbour relatedness affected their association with mycorrhizas. Whether or not pots were inoculated, social environment did not affect juveniles in allocation to stems controlling for leaf biomass (Table S5), stem elongation (Table S6) and branchiness (Table S7). Low levels of undifferentiated soil hyphae (<1 m/g soil) were found in un-inoculated pots with juvenile plants (Fig 2, white bars), possibly saprobes feeding on the cellulose pots. There was no difference in soil hyphal colonization across neighbour treatments in the un-inoculated pots (Fig 2, white bars). However, in inoculated pots, siblings had more soil hyphae than solitary plants (averaged across four pots), which in turn had more than strangers (Fig 2, black bars). There were no significant differences in hyphal root colonization between kin, strangers and solitary plants (*P = *0.9679).

**Figure 3:**
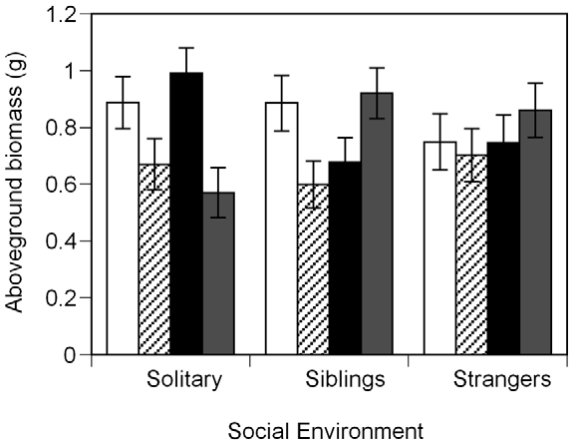
Effect of mycorrhizal inoculation, P level and social environment on aboveground biomass for juvenile *A. artemisiifolia L* plants. Plants were grown in shared pots (siblings or strangers) or solitary (alone) (*n = *606). Groups of four plants were either un-inoculated and given high P fertilizer (white bars), un-inoculated and given low P fertilizer (striped bars), inoculated and given high P fertilizer (black bars) or inoculated and given low P fertilizer (grey bars). Spores of *G. intraradices* were used to inoculate pots. Data presented are the back-transformed lsmeans of log(aboveground biomass +0.5). Error bars represent ±1 s.e.m.

Plants in the low P treatment increased allocation to fine roots relative to leaf mass (*F*_1,165_ = 29.61, *P*<0.0001). However, the effect of P on aboveground biomass depended on whether plants were in solitary or shared pots (Table 3). Solitary plants had the highest aboveground biomass when grown with high P, regardless of inoculation treatment (Fig 3). For plants grown with strangers, aboveground biomass did not differ across treatment combinations with no mycorrhizas × P level interaction (Fig 3). Plants grown with siblings demonstrated a more complex mycorrhizas × P level interaction, with the largest plants from either the un-inoculated, high P or inoculated, low P treatment combinations (Fig 3). High P plants had greater stem elongation than low P plants in the absence of mycorrhizas but there was no difference across P levels for inoculated plants (mycorrhizas × P level interaction; Table S6). In plants inoculated with mycorrhizas, we found more vesicles colonizing the roots for a given fine root mass in the high P treatment compared to low P (*F*_1,79_ = 5.80, *P = *0.0184, Fig S2).

**Figure 4:**
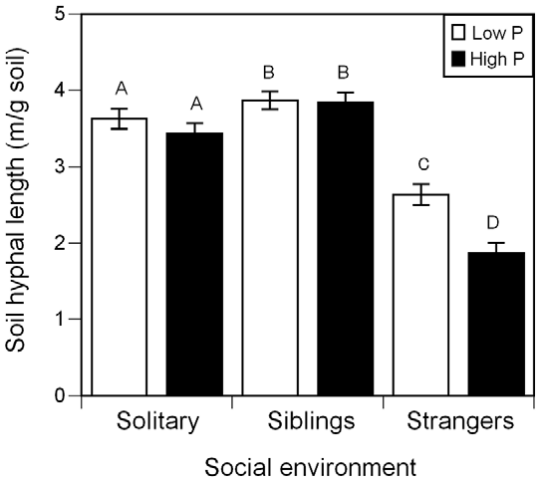
Effect of nutrient level and social environment on soil hyphal length for juvenile *A. artemisiifolia L* plants. Groups of four were solitary, siblings or strangers and all were inoculated with *G. intraradices* (*n* = 93). Solitary and sibling groups had high soil hyphal length in both high and low P, but strangers had low soil hyphal length in high P and increased soil hyphal length in low P (social environment × P level interaction *P = *0.0338). Log fine root mass was the covariate and had no effect. White bars represent groups that received low P fertilizer, and black bars are groups in high P. Means that did not differ significantly at P<0.05 are represented by the same letter. Error bars represent ±1 s.e.m.

Soil hyphal length increased in low P (main effect P level, Table 2) but it was entirely due to the difference between high and low P in stranger groups. We found that soil hyphal responses to P level depended on relatedness of the plant group (kin vs. stranger × mycorrhizas × P level, Table 2). Sibling and solitary groups maintained high hyphal length in high and low P (Fig 4). By contrast, strangers in low P had 41% more soil hyphae than strangers in high P (Fig 4). The effect of P level on arbuscule colonization also depended on social environment (*F*_2,79_ = 5.37, P = 0.0065, Fig S3); strangers inoculated with mycorrhizas in low P had more arbuscules colonizing the root than strangers in high P but there were no differences within inoculated solitary and sibling groups.

### Benefits to increased mycorrhizal association

**Figure 5:**
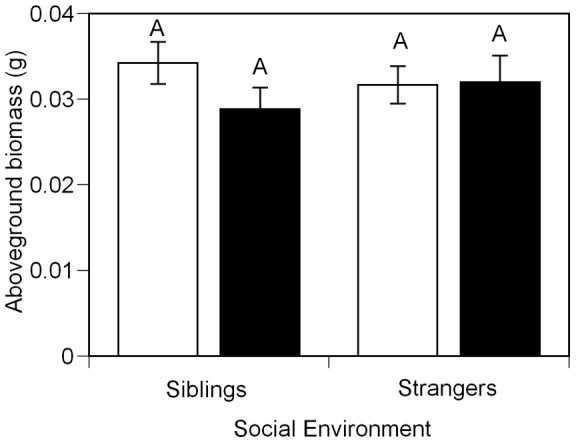
Effect of mycorrhizal inoculation and social environment on aboveground biomass for seedling *A. artemisiifolia L* plants. Plants were grown in pairs in shared pots (*n* = 238). Pairs were either un-inoculated (white bars) or inoculated (black bars) with *G. intraradices.* Data presented are the back-transformed lsmeans of log(aboveground biomass +1). Means that did not differ significantly at P<0.05 are represented by the same letter. Error bars represent ±1 s.e.m.

**Figure 6:**
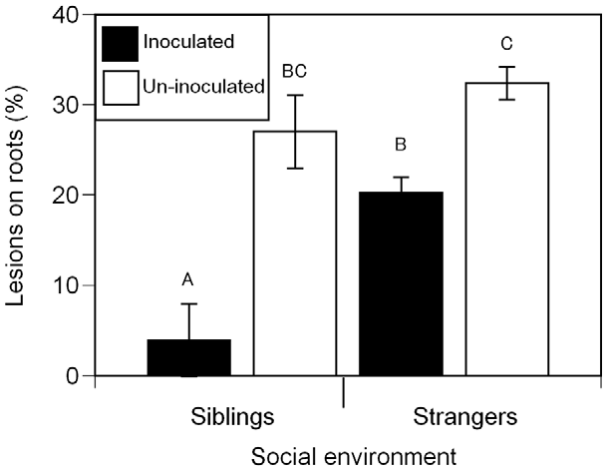
Percent of the root afflicted by lesions in pairs of *A. artemisiifolia L* seedlings. Black bars represent plants inoculated with *G. intraradices* and white bars represent un-inoculated plants (*n* = 220). Means that did not differ significantly at P<0.05 are represented by the same letter. Error bars represent ±1 s.e.m.

**Figure 7:**
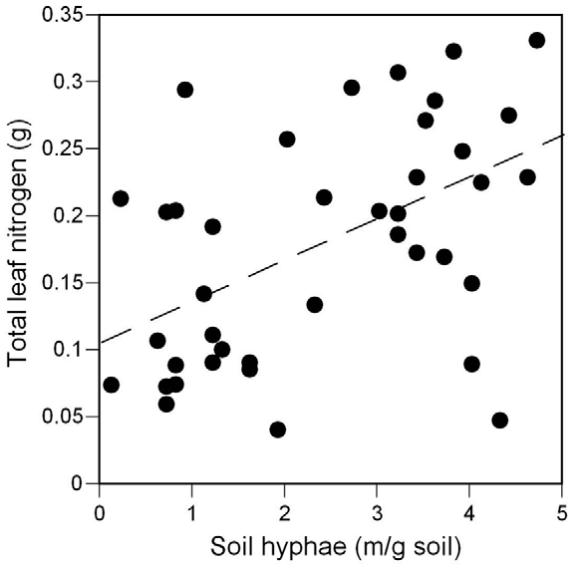
Effect of soil hyphae on leaf N in juvenile *A. artemisiifolia L* plants. Total leaf N is estimated from leaf mass × leaf N concentration. Soil hyphal length was measured in meters per gram of soil (m/g soil), for a subsample of plants grown in low P, with or without *G. intraradices*, with siblings or strangers (*n* = 40). The correlation coefficient is 0.47612 with *P = *0.0019. Equation of the line is: total nitrogen = 0.1088+.0285(soil hyphal length).

Although a field study found positive effects of *G. intraradices* on ragweed after 72 days of growth [43], we found no effects of mycorrhizas on biomass in either seedlings (Table 1, Fig 5) or juveniles (Table 3, Fig 3), possibly because plants were grown with relatively abundant nutrients [3]. However, finding a lack of effect of mycorrhizas on biomass also indicates that inoculated plants were not parasitized by the fungal partner. In the seedling experiment, plants inoculated with mycorrhizas had significantly fewer lesions on their roots compared to plants without mycorrhizas in the same social environment (black bars vs. white bars, Fig 6). Across social environments, siblings inoculated with mycorrhizas had markedly fewer lesions on their roots compared to inoculated strangers (black bars, Fig 6). In the juvenile experiment, total plant biomass was not affected by the mycorrhizal treatment (P<0.2538). However, groups in inoculated pots had significantly higher total leaf N estimated from the product of leaf mass and leaf N concentration (percent by mass), than plants in un-inoculated pots (inoculated mean =  0.2144, SE = 0.0151; un-inoculated mean = 0.1224, SE = 0.0187;P<0.0007). Total leaf N was positively correlated with soil hyphal length (correlation coefficient = 0.47612; P<0.0019; Fig 7), suggesting that larger mycorrhizal networks were associated with improved plant N uptake.

## Discussion/Conclusion

We provide the first evidence there is plant kin recognition, *i.e.*, plasticity to relatedness of neighbours, in the mycorrhizal symbiosis, and that siblings can benefit each other through increased mycorrhizal association. Though no evidence of kin recognition was found in the plants themselves, mycorrhizal colonization and growth may be considered an extended phenotype that responds to the host environmental conditions, including the relatedness of the plant group. In young seedlings, arbuscule and root hyphal colonization responded to relatedness, and pairs of siblings had fewer root lesions than strangers. Juvenile plant investment in the mycorrhizal network depended on the social environment and the nutrient conditions, which translated into a nutritional benefit for plant groups with more soil hyphae. Interestingly, we also found increased fungal colonization in low P, as predicted by the biological market model.

The mycorrhizal response to siblings is supported by kin selection theory. In the presence of likely cheaters, *i.e.*, strangers, mycorrhizal colonization and growth were lower, whereas in the absence of likely cheaters, *i.e.,* in solitary or sibling groups, mycorrhizal colonization and growth was greater. Although we found this pattern in both seedling and juvenile experiments, the mycorrhizal structures that responded were different. In seedlings, we found more arbuscules and root hyphae in siblings than in stranger pairs. Arbuscules, the sites of nutrient exchange, are relatively short-lived (4–10 days) [1] and thus the level of root colonization could easily change over a plant's lifetime. In juveniles we found more soil hyphal colonization in groups of siblings compared to strangers. Early in life, the net benefit of associating with mycorrhizas is lower compared to later on because the seedling is donating carbon to the fungal partner that could otherwise be used for its own growth and defence [4]. However, higher root colonization at the seedling stage can have benefits for nutrient uptake at the juvenile and adult stages [45], which could translate into increased final fitness. This benefit would be even greater if plants were colonizing a CMN connected with related individuals, potentially increasing their inclusive fitness. Our findings from both experiments support this idea since sibling pairs had greater arbuscular colonization than strangers, and at a later life-stage, groups of siblings had increased soil hyphae.

Greater soil hyphal length in juvenile sibling groups implies that the plants growing with siblings actively increased their investment in the mycorrhizal association. Consistent with predictions from the social good model, siblings appeared to contribute more to the symbiosis compared to strangers by supporting increased fungal growth in the soil. Plants have the ability to control their carbohydrate donations to fungi, preferentially allocating carbon to more beneficial fungal partners over more parasitic ones [11], leading to increased fungal fitness [13], so it is also possible that they could preferentially allocate to a CMN attached to siblings versus one attached to strangers. Similar to previous research [44], we found no trade-offs between fungal traits (Table S8), supporting the argument that soil hyphae is an indicator of plant contribution to fungal growth. The larger network size in groups of related plants implies that the fungus benefits from plant kin selection. Thus, the plant neighbourhood may be a key influence on the fitness of the fungal partner.

It might be argued that the increased mycorrhizal association in sibling groups is evidence that the fungal partner can more effectively exploit genetically similar groups. In this parasitism hypothesis, finding more arbuscules in seedlings and more soil hyphae in juveniles can be interpreted as fungal success in sibling groups. Evidence against this parasitism hypothesis would be the observation that plants benefit from increased fungal colonization. We measured two potential short-term benefits that can specifically be attributed to mycorrhizas. First, we found fewer lesions with seedlings associating with mycorrhizas, with sibling pairs having significantly fewer than strangers. This decrease in general lesion number indicates an overall protective effect of mycorrhizas on young seedling roots, suggesting that there are early benefits for siblings who increase their association with mycorrhizal fungi at the seedling stage. The lesions observed on the roots from our seedling study could have come from various sources including fungal pathogens, parasites and root nematodes. However, mycorrhizal fungi are known to benefit plants by protecting them against root lesions through a variety of mechanisms, including competition between pathogens and AM-fungi (reviewed in [1]). The second observation against the parasitism hypothesis is that our data suggests inoculated pots of juvenile plants had higher total leaf N, a result that is consistent with the generally positive effects of soil hyphal length on plant nutrient status [44]. N and P acquisition are often correlated and N is typically the most important limiting nutrient for plant growth [46], and pollen and seed production [47]. Therefore, juvenile plants in sibling groups may have had improved nutrient acquisition ability through an extended mycorrhizal network resulting from their increased investment. Thus for both seedlings and juveniles, there are short-term benefits to having greater mycorrhizal association which could result in higher survival and fecundity for plants grown with siblings. This is further evidence supporting the argument for kin selection acting on the ragweed-mycorrhizal symbiosis.

Our results suggest that juvenile siblings invested carbon in mycorrhizas even at high P, when the mutualism is likely less necessary for P uptake. Despite a common prediction that plants will have higher association with mycorrhizal fungi in low P [5], we found that only strangers had this response. In contrast, siblings and solitary plants maintained consistently higher levels of soil hyphae across P levels. A high level of investment in mycorrhizas, despite high P, could provide multiple benefits including bet hedging against future demand for nutrients, increased water acquisition, and pathogen defense [1], all of which could increase the chances of survival and, therefore, final fitness. These benefits could increase one's indirect fitness when attached to the same CMN as relatives.

We were able to reject our alternative hypotheses about the causes of mycorrhizal and plant benefit differences across social environments. Previous studies of plant recognition have found phenotypic plasticity to neighbours in nutrient acquisition traits, including fine roots [27]–[30]. Consequently, one alternative hypothesis is that changes in plant morphology induced by kin recognition caused the differences found in mycorrhizas. However, in neither experiment were there shifts in biomass allocation or aboveground morphological changes in response to social environment. Therefore, plant morphological responses to social environment were not confounded with responses seen in the fungal partner. The only trait showing any social environment interactions was log aboveground biomass in juveniles. Here, the differences among families in solitary vs. shared effects and in kin vs. stranger effects (social environment × family, Table 3, Fig S4) were the consequence of more variance among families in stranger than kin or solitary conditions. In the seedling study, we found no effect of family on fungal structures typically associated with strength of the mutualism, arbuscules (P<0.8706) and hyphae (P<0.7885), allowing us to reject the hypothesis that some plant genotypes may have higher specificity for a given fungus. There were no differences in soil hyphal length between the four genotypes of juvenile plants either (Fig S5). Finding a lack of effect of family on mycorrhizal structures expected to be associated with a stronger symbiosis in both seedling and juvenile studies indicates that the increased colonization in siblings was not due to a particular family having stronger associations with the fungal genotype used in either experiment. We also investigated whether the differences in soil mycorrhizas were the result of soil hyphae being correlated with biomass of the root sample used for fungal quantification, coupled with systematic differences in root biomass between social environments. *Post hoc* analysis revealed no relationship between root sample mass and soil hyphal length (Fig S6). Above- and belowground biomasses were strongly positively correlated with each other but not with any of the fungal traits. Root hyphal colonization and arbuscular colonization were negatively correlated (P<0.0278). No other fungal traits were correlated (Table S8).

Previous research in *Arabidopsis thaliana* has demonstrated that the mechanism for plant kin recognition involves root exudates [27]. We hypothesize that ragweed also uses root exudates to recognize the identity of surrounding plants. If ragweed recognizes that it is growing near siblings and it is also attached to a mycorrhizal fungal partner, it may altruistically donate more carbon to the fungal partner. Kin selection would favour this increased donation since the benefits that could be provided to neighbouring kin would increase the focal individual's inclusive fitness. Alternatively, if a focal individual recognized its neighbours as strangers, it could avoid costly contributions to the CMN that would benefit non-relatives and provide no inclusive fitness rewards.

In conclusion, mycorrhizal colonization and growth was highest in sibling groups, supporting predictions from social good theory that kin selection can stabilize a mutualism [12]. Though a previous study provided evidence that plants benefit from population level specificity to soil fungal communities [37], here we demonstrate that the mycorrhizal symbiosis is also affected by plant kin recognition. Low nutrient availability is known to favour mycorrhizal colonization [48]. However, our results indicate that plant neighborhood may determine the extent of this nutrient effect, since sibling plants invested more in the mycorrhizal network regardless of P level. Moreover, the effect of social environment on soil hyphae was much greater than the effect of increased P. Thus, even in high P where mutualism break down is predicted, plant kin selection may allow fungal populations to persist. Though these results were found in greenhouse studies, natural population structure created through limited seed dispersal can also generate proximity among siblings [49], suggesting that kin recognition could be an important mechanism that reinforces the ancient mutualism between plants and fungi.

## Supporting Information

Figure S1 Effect of life stage on mycorrhizal root colonization of *A. artemisiifolia L* roots. Inoculated plants had vesicles (black bars) and arbuscules (white bars) colonizing the roots of both seedlings and juveniles. Log fine root mass did not affect fungal colonization. Un-inoculated plants were not included in this graph because no arbuscules or vesicles were found in soil samples from un-inoculated pots. Error bars represent ±1 s.e.m.
(TIF)

Figure S2 Effect of nutrient level on vesicle colonization on inoculated juvenile *A. artemisiifolia L* roots. Groups of four plants were inoculated with *G. intraradices*. Un-inoculated plants were not included in this graph because no vesicles were found colonizing their roots. Inoculated plants had more vesicles in high P (mycorrhizas × P level interaction, *P = *0.0177). Log fine root mass is the covariate and had no effect. White bars represent groups that received low P fertilizer, and black bars represent groups that received high P fertilizer. Means that did not differ significantly at P<0.05 are represented by the same letter. Error bars represent ±1 s.e.m.
(TIF)

Figure S3 Effect of nutrient level and social environment on arbuscule colonization on juvenile *A. artemisiifolia L* roots. Groups of four plants were inoculated with *G. intraradices*. Strangers responded to nutrients but solitary plants and sibling groups did not (Social environment × P level interaction *P* = 0.0065). Log fine root mass is the covariate. Plants were grown alone (solitary), with siblings or with strangers. White bars represent groups that received low P fertilizer, and black bars are groups receiving high P. Means that did not differ significantly at P<0.05 are represented by the same letter.
(TIF)

Figure S4 Effect of family on aboveground biomass for juvenile *A. artemisiifolia L* plants. Plants were grown in one of three social environments: solitary (alone), kin and stranger (*n = *606). Each symbol represents a maternal sibship (family). Closed squares: family A; closed circles: family B; open squares: family C; open circles: family D. Data presented are the back-transformed lsmeans of log(aboveground biomass +0.5). Error bars represent ±1 s.e.m.
(TIF)

Figure S5 Effect of juvenile *A. artemisiifolia L* genotype on soil hyphal length. Analysis was done on groups of four plants grown with siblings (*n* = 184). Genotypes (maternal family lines) are represented by letters A–D. There is no statistical difference among families for soil hyphal length (*P = *0.6381). Error bars represent ±1 s.e.m.
(TIF)

Figure S6 Effect of root sample mass on soil hyphal length. Root sample mass is an estimate of the dried root biomass used for fungal quantification of juvenile *A. artemisiifolia L.* plants. There is no significant relationship between soil hyphae and root sample mass (*P = *0.6911).
(TIF)

Table S1 Analysis of variance of mycorrhizal structures in ragweed seedling pairs. Only plants that were inoculated with mycorrhizal spores were analyzed. Social environment refers to kin vs. stranger. Block refers to the experimental unit. Family refers to the specific pairing of maternal sibships within each pot. Significant values are in bold.
(DOC)

Table S2 Analysis of covariance indicating stem elongation for ragweed seedling pairs. Plants were grown in pairs of either siblings or strangers, with or without mycorrhizal spores. Six maternal sibships (families) were used. Social environment and mycorrhizas refer to treatment effects. Family refers to the specific pairing of maternal sibships within each pot. Significant values are in bold.
(DOC)

Table S3 Analysis of covariance for leaf:stem allocation for ragweed seedling pairs. Plants were grown in pairs of either siblings or strangers, with or without mycorrhizal spores. Six maternal sibships (families) were used. Social environment and mycorrhizas refer to treatment effects. Family refers to the specific pairing of families within each pot. Significant values are in bold.
(DOC)

Table S4 Analysis of covariance showing root:shoot allocation for ragweed seedling pairs. Plants were grown in pairs of either siblings or strangers, with or without mycorrhizas. Social environment and mycorrhizas refer to treatment effects. Family refers to maternal sibship. Significant values are in bold.
(DOC)

Table S5 Analysis of covariance showing stem:leaf allocation for groups of ragweed juveniles. Plants were grown in groups of four. Social environment, mycorrhizas and P level refer to treatment effects. Family refers to specific maternal sibships within each group. Log stem is log(stem biomass +1) and log leaf is log(leaf biomass +1). Significant values are in bold.
(DOC)

Table S6 Analysis of covariance showing stem elongation for groups of ragweed juveniles. Plants were grown in groups of four. Social environment, mycorrhizas and nutrient level refer to treatment effects. Family refers to specific maternal sibships within each group. Significant values are in bold.
(DOC)

Table S7 Analysis of covariance indicating branchiness for groups of ragweed juveniles. Branch number:log aboveground biomass is a metric of branchiness. Log above is log(aboveground biomass +0.5). Social environment, mycorrhizas and P level refer to treatment effects. Family refers to specific maternal sibships within each group. Significant values are in bold.
(DOC)

Table S8 Correlation matrix for juvenile ragweed plants. Only plants inoculated with *G. intraradices* were used in this analysis. Spearman correlation was used. Significant values are in bold.
(DOC)
