# Supplementary figures and images for: Plant Kin Recognition Enhances Abundance of Symbiotic Microbial Partner
Source: PLoS One. 2012 Sep 28;7(9):e45648. doi: 10.1371/journal.pone.0045648 (PMC3460938; doi:10.1371/journal.pone.0045648)

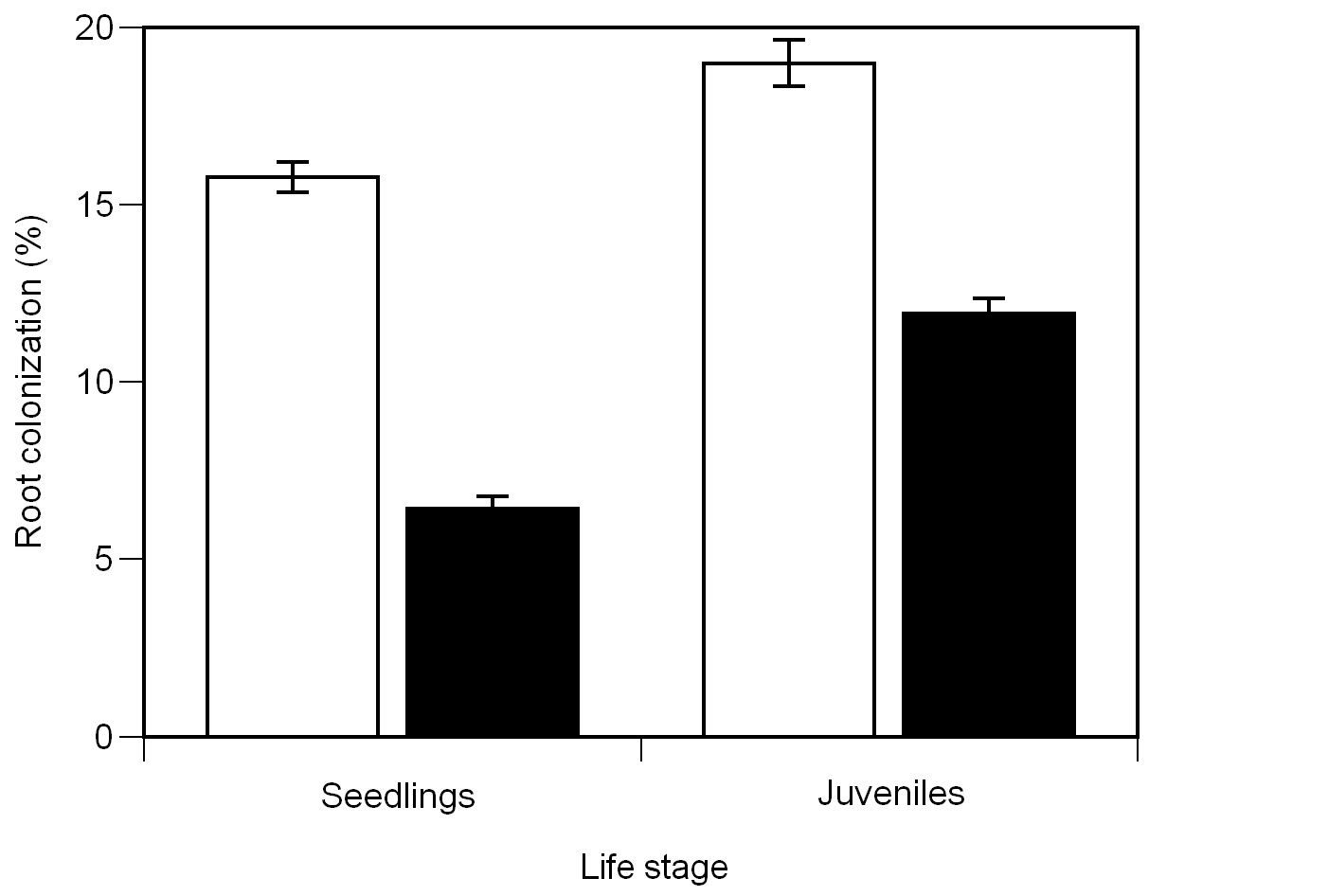

Supplement: Figure S1 — Effect of life stage on mycorrhizal root colonization of A. artemisiifolia L roots. Inoculated plants had vesicles (black bars) and arbuscules (white bars) colonizing the roots of both seedlings and juveniles. Log fine root mass did not affect fungal colonization. Un-inoculated plants were not included in this graph because no arbuscules or vesicles were found in soil samples from un-inoculated pots. Error bars represent ±1 s.e.m. (TIF) [file pone.0045648.s001.tif]

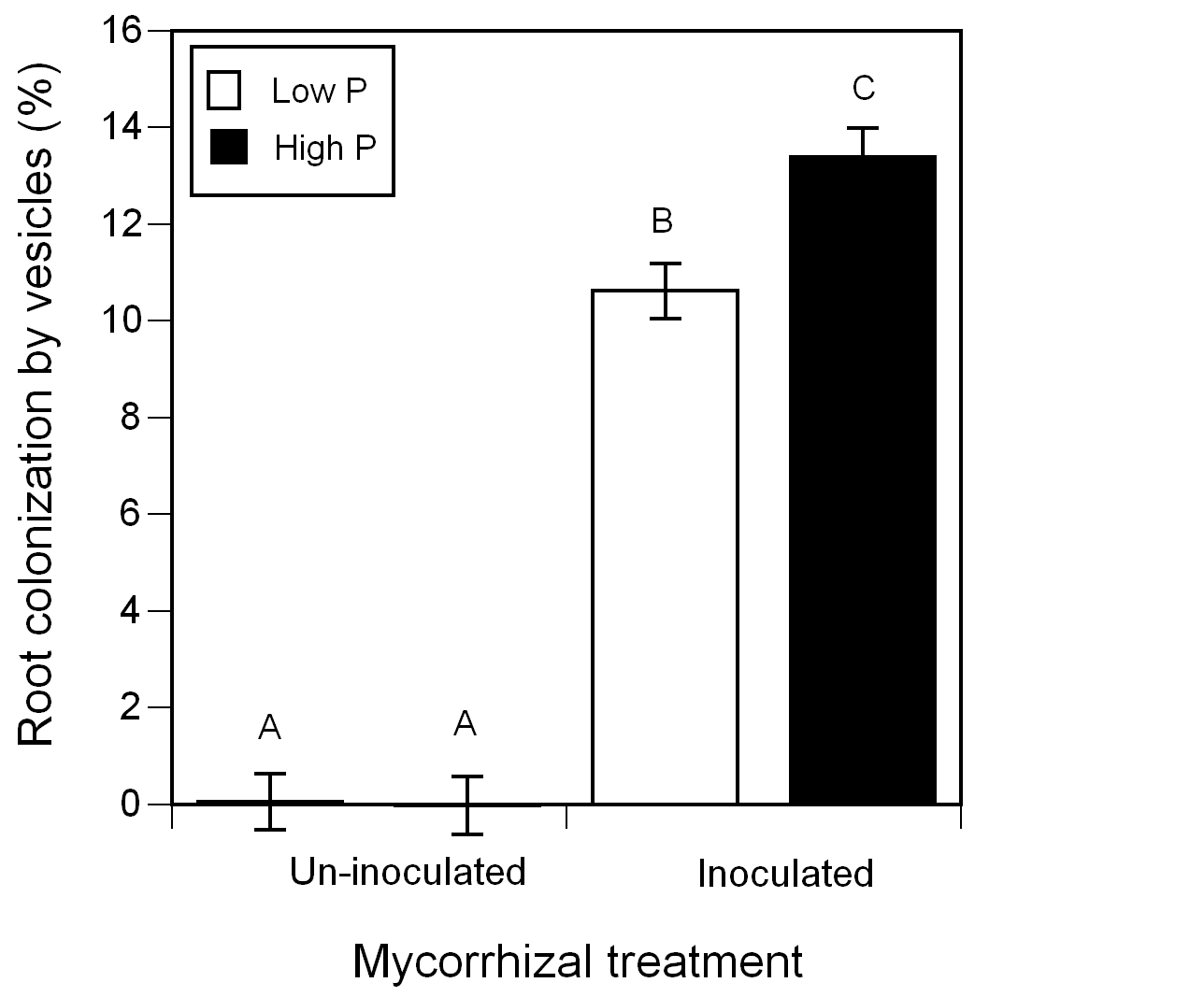

Supplement: Figure S2 — Effect of nutrient level on vesicle colonization on inoculated juvenile A. artemisiifolia L roots. Groups of four plants were inoculated with G. intraradices. Un-inoculated plants were not included in this graph because no vesicles were found colonizing their roots. Inoculated plants had more vesicles in high P (mycorrhizas × P level interaction, P = 0.0177). Log fine root mass is the covariate and had no effect. White bars represent groups that received low P fertilizer, and black bars represent groups that received high P fertilizer. Means that did not differ significantly at P<0.05 are represented by the same letter. Error bars represent ±1 s.e.m. (TIF) [file pone.0045648.s002.tif]

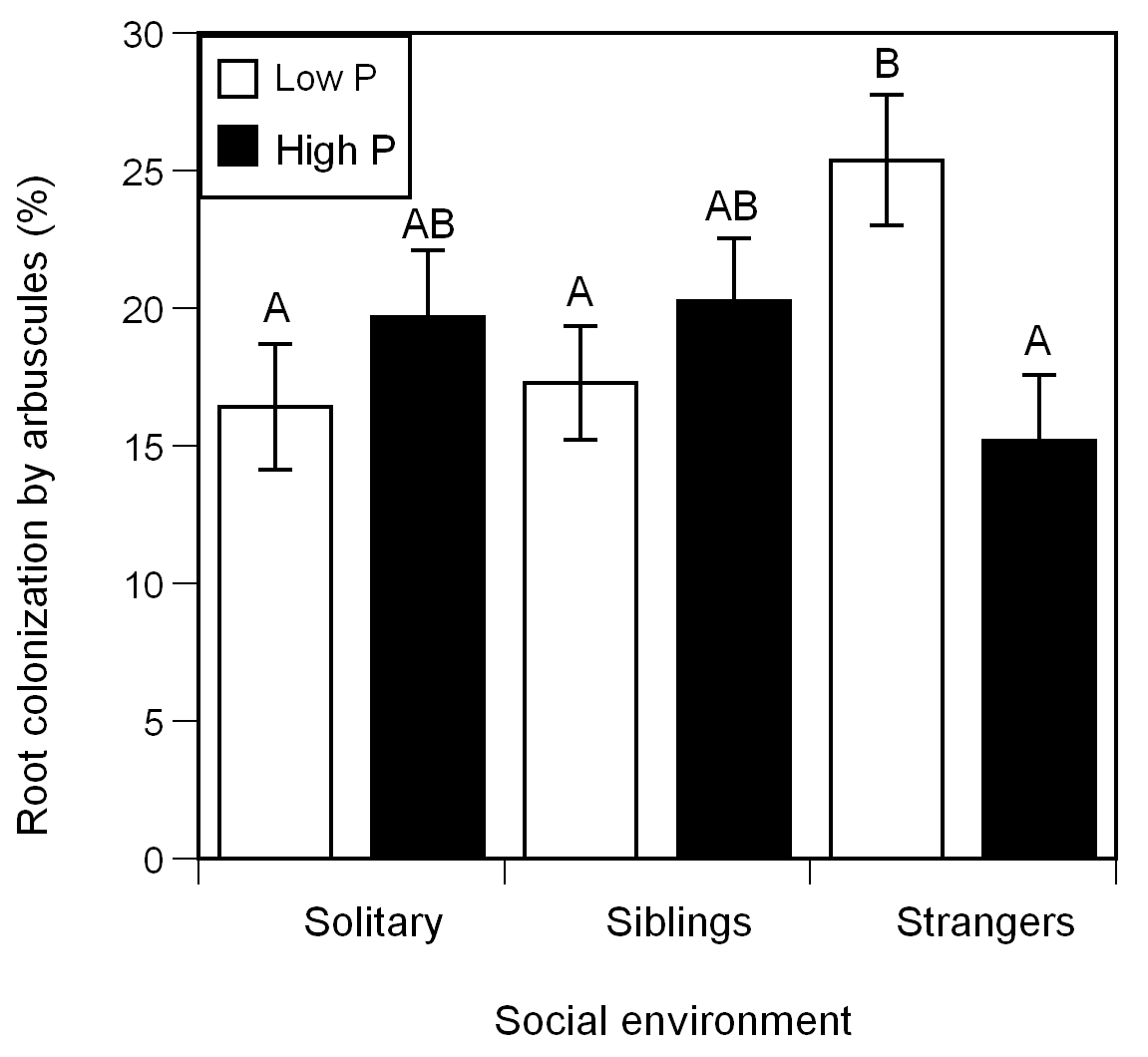

Supplement: Figure S3 — Effect of nutrient level and social environment on arbuscule colonization on juvenile A. artemisiifolia L roots. Groups of four plants were inoculated with G. intraradices. Strangers responded to nutrients but solitary plants and sibling groups did not (Social environment × P level interaction P = 0.0065). Log fine root mass is the covariate. Plants were grown alone (solitary), with siblings or with strangers. White bars represent groups that received low P fertilizer, and black bars are groups receiving high P. Means that did not differ significantly at P<0.05 are represented by the same letter. (TIF) [file pone.0045648.s003.tif]

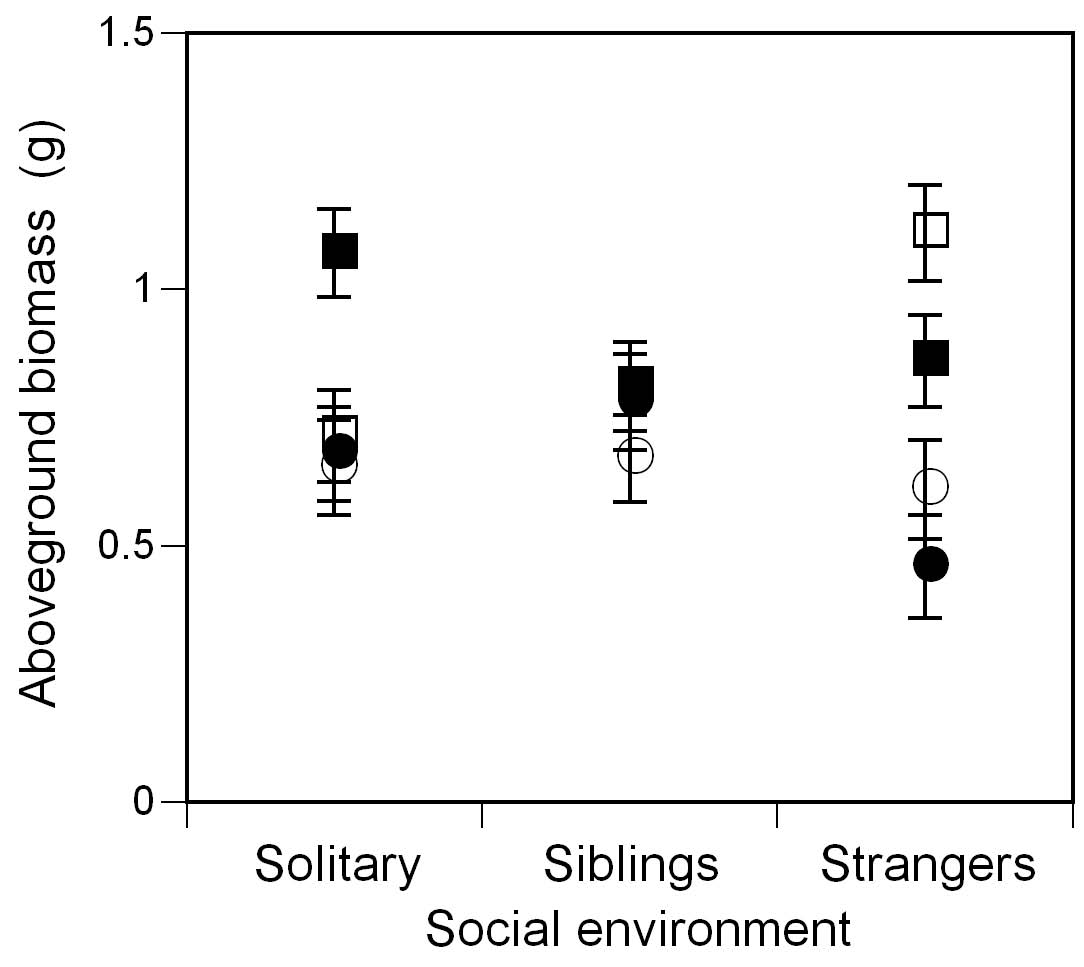

Supplement: Figure S4 — Effect of family on aboveground biomass for juvenile A. artemisiifolia L plants. Plants were grown in one of three social environments: solitary (alone), kin and stranger (n = 606). Each symbol represents a maternal sibship (family). Closed squares: family A; closed circles: family B; open squares: family C; open circles: family D. Data presented are the back-transformed lsmeans of log(aboveground biomass +0.5). Error bars represent ±1 s.e.m. (TIF) [file pone.0045648.s004.tif]

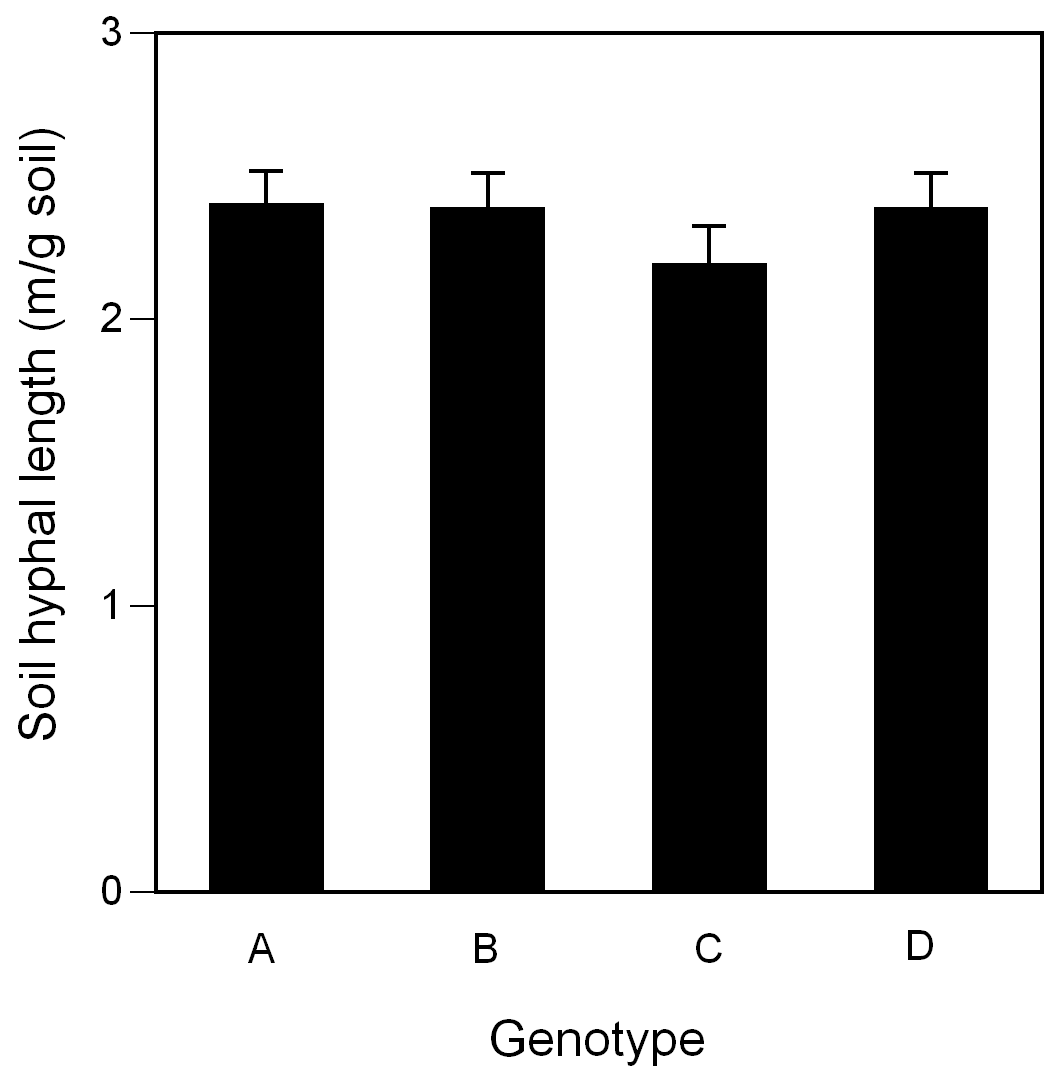

Supplement: Figure S5 — Effect of juvenile A. artemisiifolia L genotype on soil hyphal length. Analysis was done on groups of four plants grown with siblings (n = 184). Genotypes (maternal family lines) are represented by letters A–D. There is no statistical difference among families for soil hyphal length (P = 0.6381). Error bars represent ±1 s.e.m. (TIF) [file pone.0045648.s005.tif]

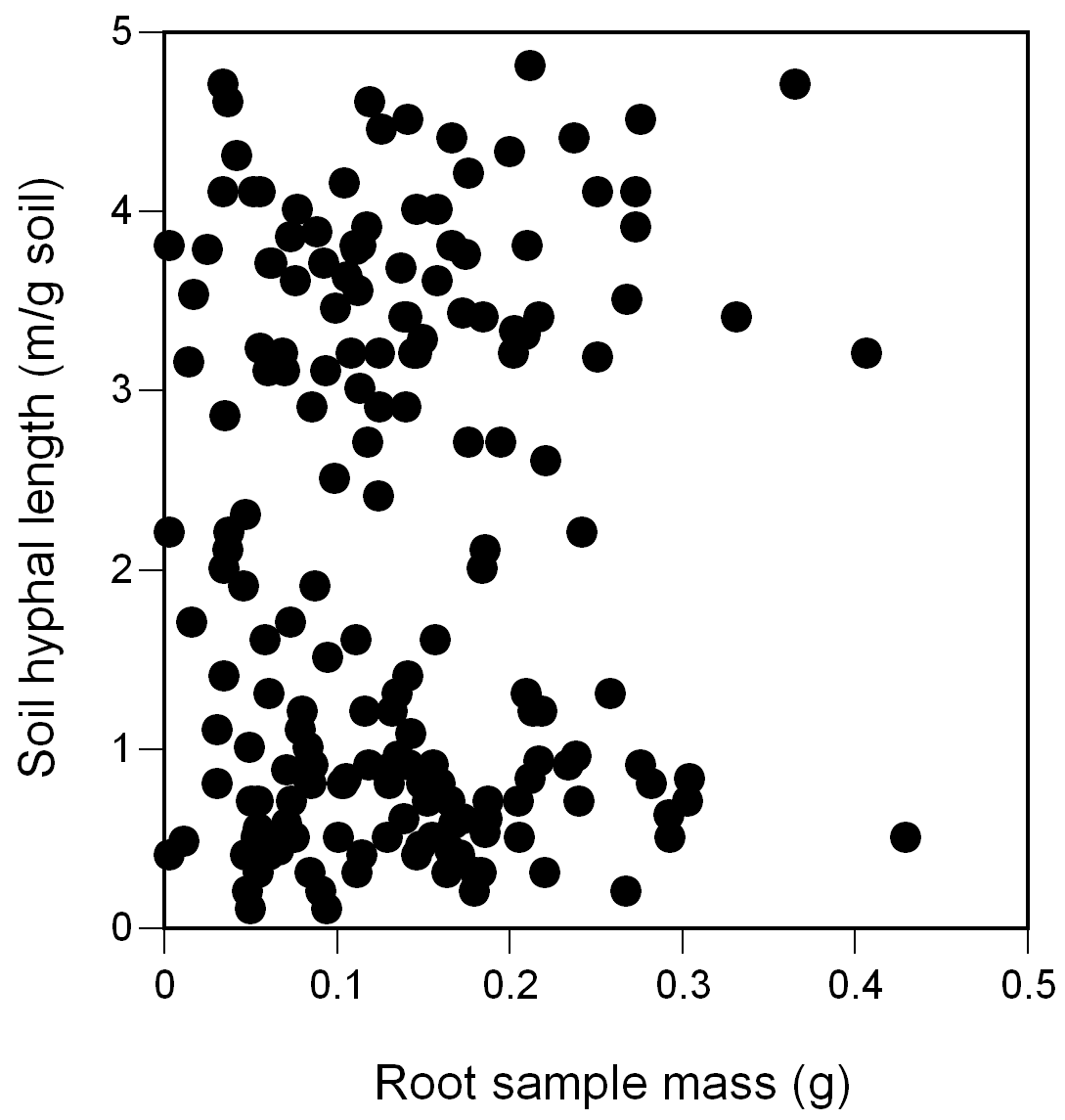

Supplement: Figure S6 — Effect of root sample mass on soil hyphal length. Root sample mass is an estimate of the dried root biomass used for fungal quantification of juvenile A. artemisiifolia L. plants. There is no significant relationship between soil hyphae and root sample mass (P = 0.6911). (TIF) [file pone.0045648.s006.tif]
