# Supplementary material for: Plant Kin Recognition Enhances Abundance of Symbiotic Microbial Partner
Source: PLoS One. 2012 Sep 28;7(9):e45648. doi: 10.1371/journal.pone.0045648 (PMC3460938; doi:10.1371/journal.pone.0045648)
Supplement: Table S1 — Analysis of variance of mycorrhizal structures in ragweed seedling pairs. Only plants that were inoculated with mycorrhizal spores were analyzed. Social environment refers to kin vs. stranger. Block refers to the experimental unit. Family refers to the specific pairing of maternal sibships within each pot. Significant values are in bold. (DOC) [file pone.0045648.s007.doc]

| Table S1: Analysis of variance of mycorrhizal structures in ragweed seedling pairs. | | | | | | | | | |
| --- | --- | --- | --- | --- | --- | --- | --- | --- | --- |
|  | Arbuscules | | | Root hyphae | | | Vesicles | | |
| Source | DF | F | *P* | DF | F | *P* | DF | F | *P* |
| Social environment | 1 | 16.70 | **<0.0001** | 1 | 48.65 | **<0.0001** | 1 | 0.84 | 0.3620 |
| Block | 5 | 3.35 | 0.0084 | 5 | 1.83 | 0.1162 | 5 | 1.43 | 0.2208 |
| SocialEnv × block | 5 | 3.84 | 0.0035 | 5 | 1.25 | 0.2916 | 5 | 1.10 | 0.3656 |
| Family | 14 | 3.85 | **<0.0001** | 14 | 3.33 | **0.0003** | 14 | 2.75 | **0.0022** |
