# Supplementary material for: Plant Kin Recognition Enhances Abundance of Symbiotic Microbial Partner
Source: PLoS One. 2012 Sep 28;7(9):e45648. doi: 10.1371/journal.pone.0045648 (PMC3460938; doi:10.1371/journal.pone.0045648)
Supplement: Table S2 — Analysis of covariance indicating stem elongation for ragweed seedling pairs. Plants were grown in pairs of either siblings or strangers, with or without mycorrhizal spores. Six maternal sibships (families) were used. Social environment and mycorrhizas refer to treatment effects. Family refers to the specific pairing of maternal sibships within each pot. Significant values are in bold. (DOC) [file pone.0045648.s008.doc]

| Table S2: Analysis of covariance indicating stem elongation for ragweed seedling pairs. | | | |
| --- | --- | --- | --- |
|  | Height (cm) | | |
| Source | DF | F | *P* |
| Stem biomass (g) | 1 | 322.36 | **<0.0001** |
| Mycorrhizas | 1 | 2.04 | 0.1549 |
| Social environment | 1 | 0.12 | 0.7313 |
| Family | 5 | 5.51 | **<0.0001** |
| Myc × SocialEnv | 1 | 0.04 | 0.8476 |
| Myc × Fam | 5 | 0.40 | 0.8452 |
| SocialEnv × Fam | 5 | 2.20 | 0.0551 |
| Myc × SocialEnv × Fam | 5 | 1.00 | 0.4185 |
