# Supplementary material for: Plant Kin Recognition Enhances Abundance of Symbiotic Microbial Partner
Source: PLoS One. 2012 Sep 28;7(9):e45648. doi: 10.1371/journal.pone.0045648 (PMC3460938; doi:10.1371/journal.pone.0045648)
Supplement: Table S3 — Analysis of covariance for leaf:stem allocation for ragweed seedling pairs. Plants were grown in pairs of either siblings or strangers, with or without mycorrhizal spores. Six maternal sibships (families) were used. Social environment and mycorrhizas refer to treatment effects. Family refers to the specific pairing of families within each pot. Significant values are in bold. (DOC) [file pone.0045648.s009.doc]

| Table S3: Analysis of covariance for leaf:stem allocation for ragweed seedling pairs. | | | |
| --- | --- | --- | --- |
|  | Leaf biomass (g) | | |
| Source | DF | F | *P* |
| Stem biomass (g) | 1 | 237.56 | **<0.0001** |
| Mycorrhizas | 1 | 0.03 | 0.8693 |
| Social environment | 1 | 0.67 | 0.4141 |
| Family | 5 | 11.56 | **<0.0001** |
| Myc × SocialEnv | 1 | 0.41 | 0.5237 |
| Myc × Fam | 5 | 0.09 | 0.9945 |
| SocialEnv × Fam | 5 | 1.41 | 0.2236 |
| Myc × SocialEnv × Fam | 5 | 1.18 | 0.3211 |
