# Supplementary material for: Plant Kin Recognition Enhances Abundance of Symbiotic Microbial Partner
Source: PLoS One. 2012 Sep 28;7(9):e45648. doi: 10.1371/journal.pone.0045648 (PMC3460938; doi:10.1371/journal.pone.0045648)
Supplement: Table S4 — Analysis of covariance showing root:shoot allocation for ragweed seedling pairs. Plants were grown in pairs of either siblings or strangers, with or without mycorrhizas. Social environment and mycorrhizas refer to treatment effects. Family refers to maternal sibship. Significant values are in bold. (DOC) [file pone.0045648.s010.doc]

| Table S4: Analysis of covariance showing root:shoot allocation for ragweed seedling pairs. | | | |
| --- | --- | --- | --- |
|  | Belowground biomass (g) | | |
| Source | DF | F | *P* |
| Aboveground biomass (g) | 1 | 504.78 | **<0.0001** |
| Mycorrhizas | 1 | 0.28 | 0.5943 |
| Social environment | 1 | 0.54 | 0.4630 |
| Myc × SocialEnv | 1 | 0.02 | 0.8885 |
