# Supplementary material for: Plant Kin Recognition Enhances Abundance of Symbiotic Microbial Partner
Source: PLoS One. 2012 Sep 28;7(9):e45648. doi: 10.1371/journal.pone.0045648 (PMC3460938; doi:10.1371/journal.pone.0045648)
Supplement: Table S5 — Analysis of covariance showing stem:leaf allocation for groups of ragweed juveniles. Plants were grown in groups of four. Social environment, mycorrhizas and P level refer to treatment effects. Family refers to specific maternal sibships within each group. Log stem is log(stem biomass +1) and log leaf is log(leaf biomass +1). Significant values are in bold. (DOC) [file pone.0045648.s011.doc]

| Table S5: Analysis of covariance showing stem:leaf allocation for groups of ragweed juveniles. | | | |
| --- | --- | --- | --- |
|  | Log stem | | |
| Source | DF | F | *P* |
| Log leaf | 1 | 4681.05 | **<0.0001** |
| Social environment | 2 | 0.34 | 0.7105 |
| Mycorrhizas | 1 | 2.21 | 0.1371 |
| P level | 1 | 0.80 | 0.3715 |
| Family | 3 | 8.31 | **<0.0001** |
| Myc × SocialEnv | 2 | 0.33 | 0.7188 |
| Myc × Fam | 3 | 0.18 | 0.9132 |
| Myc × P level | 1 | 0.57 | 0.4518 |
| SocialEnv × Fam | 6 | 0.92 | 0.4835 |
| SocialEnv × P level | 2 | 0.77 | 0.4646 |
| P level × Fam | 3 | 0.91 | 0.4336 |
| SocialEnv × Myc × Fam | 6 | 0.85 | 0.5346 |
| Myc × P × Fam | 3 | 2.20 | 0.0868 |
| SocialEnv × P × Fam | 6 | 1.02 | 0.4086 |
| SocialEnv × Myc × P | 2 | 0.77 | 0.4617 |
| SocialEnv × Myc × P × Fam | 6 | 0.51 | 0.8015 |
| Block | 5 | 1.78 | 0.1160 |
