# Supplementary material for: Plant Kin Recognition Enhances Abundance of Symbiotic Microbial Partner
Source: PLoS One. 2012 Sep 28;7(9):e45648. doi: 10.1371/journal.pone.0045648 (PMC3460938; doi:10.1371/journal.pone.0045648)
Supplement: Table S6 — Analysis of covariance showing stem elongation for groups of ragweed juveniles. Plants were grown in groups of four. Social environment, mycorrhizas and nutrient level refer to treatment effects. Family refers to specific maternal sibships within each group. Significant values are in bold. (DOC) [file pone.0045648.s012.doc]

| Table S6: Analysis of covariance showing stem elongation for groups of ragweed juveniles. | | | |
| --- | --- | --- | --- |
|  | Height (cm) | | |
| Source | DF | F | *P* |
| Stem biomass (g) | 1 | 385.00 | **<0.0001** |
| Stem × stem | 1 | 160.60 | **<0.0001** |
| Social environment | 2 | 2.80 | 0.0616 |
| Mycorrhizas | 1 | 0.73 | 0.3939 |
| P level | 1 | 1.44 | 0.2299 |
| Family | 3 | 5.69 | **0.0008** |
| Myc × SocialEnv | 2 | 1.68 | 0.1875 |
| Myc × Fam | 3 | 0.20 | 0.8992 |
| Myc × P level | 1 | 7.91 | **0.0051** |
| SocialEnv × Fam | 6 | 1.48 | 0.1843 |
| SocialEnv × P level | 2 | 2.64 | 0.0725 |
| P level × Fam | 3 | 2.36 | 0.0710 |
| SocialEnv × Myc × Fam | 6 | 0.55 | 0.7713 |
| Myc X P × Fam | 3 | 0.65 | 0.5823 |
| SocialEnv × P × Fam | 6 | 1.69 | 0.1202 |
| SocialEnv × Myc × P | 2 | 0.91 | 0.4018 |
| SocialEnv × Myc × P × Fam | 6 | 1.45 | 0.1916 |
| Block | 5 | 3.56 | **0.0035** |
