# Supplementary material for: Plant Kin Recognition Enhances Abundance of Symbiotic Microbial Partner
Source: PLoS One. 2012 Sep 28;7(9):e45648. doi: 10.1371/journal.pone.0045648 (PMC3460938; doi:10.1371/journal.pone.0045648)
Supplement: Table S7 — Analysis of covariance indicating branchiness for groups of ragweed juveniles. Branch number:log aboveground biomass is a metric of branchiness. Log above is log(aboveground biomass +0.5). Social environment, mycorrhizas and P level refer to treatment effects. Family refers to specific maternal sibships within each group. Significant values are in bold. (DOC) [file pone.0045648.s013.doc]

| Table S7: Analysis of covariance indicating branchiness for groups of ragweed juveniles. | | | |
| --- | --- | --- | --- |
|  | Branch number | | |
| Source | DF | F | *P* |
| Log above | 1 | 331.61 | **<0.0001** |
| Log above × log above | 1 | 89.11 | **<0.0001** |
| Social environment | 2 | 2.62 | 0.0736 |
| Mycorrhizas | 1 | 0.82 | 0.3669 |
| P level | 1 | 2.76 | 0.0975 |
| Family | 3 | 9.18 | **<0.0001** |
| Myc × SocialEnv | 2 | 0.82 | 0.4420 |
| Myc × Fam | 3 | 1.55 | 0.2006 |
| Myc × P level | 1 | 0.60 | 0.4397 |
| SocialEnv × Fam | 6 | 1.76 | 0.1059 |
| SocialEnv × P level | 2 | 1.69 | 0.1857 |
| P level × Fam | 3 | 2.46 | 0.0618 |
| SocialEnv × Myc × Fam | 6 | 1.82 | 0.0931 |
| Myc × P × Fam | 3 | 1.69 | 0.1672 |
| SocialEnv × P × Fam | 6 | 0.39 | 0.8829 |
| SocialEnv × Myc × P | 2 | 0.98 | 0.3768 |
| SocialEnv × Myc × P × Fam | 6 | 0.45 | 0.8468 |
| Block | 5 | 18.52 | **<0.0001** |
