# Supplementary material for: Plant Kin Recognition Enhances Abundance of Symbiotic Microbial Partner
Source: PLoS One. 2012 Sep 28;7(9):e45648. doi: 10.1371/journal.pone.0045648 (PMC3460938; doi:10.1371/journal.pone.0045648)
Supplement: Table S8 — Correlation matrix for juvenile ragweed plants. Only plants inoculated with G. intraradices were used in this analysis. Spearman correlation was used. Significant values are in bold. (DOC) [file pone.0045648.s014.doc]

| Table S8: Correlation matrix for juvenile ragweed plants | | | | | | |
| --- | --- | --- | --- | --- | --- | --- |
|  | Above mass | Total root | VC | HC | AC | Soil hyphae |
| Total root | **0.95701**  **<0.0001** | 1 |  |  |  |  |
| VC | 0.01632  0.8773 | -0.04623  0.6617 | 1 |  |  |  |
| HC | -0.00990  0.9254 | 0.02125  0.8406 | 0.05918  0.5731 | 1 |  |  |
| AC | -0.02896  0.7840 | -0.00969  0.9270 | -0.01234  0.9066 | **-0.22825**  **0.0278** | 1 |  |
| Soil hyphae | 0.13395  0.2030 | 0.12362  0.2404 | -0.00324  0.9754 | 0.11053  0.2915 | 0.09898  0.3452 | 1 |
